# Supplementary material for: VDUP1 Deficiency Promotes the Severity of DSS-Induced Colitis in Mice by Inducing Macrophage Infiltration
Source: Int J Mol Sci. 2023 Sep 1;24(17):13584. doi: 10.3390/ijms241713584 (PMC10487977; doi:10.3390/ijms241713584)
Supplement: Supplementary file 1 [file ijms-24-13584-s001.zip › Supplementary Figures_Final.pptx]

## Slide 1
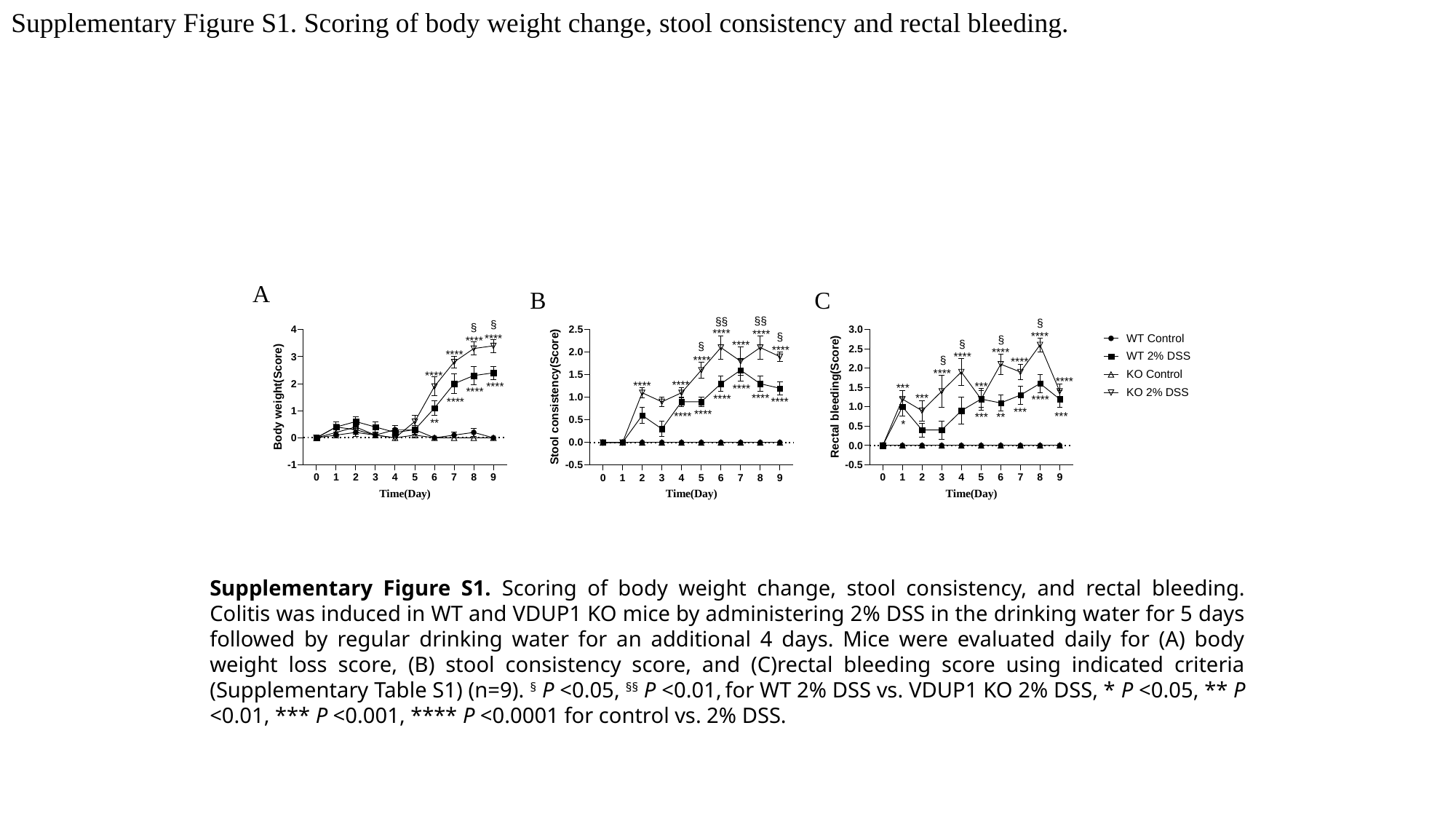

Supplementary Figure S1. Scoring of body weight change, stool consistency and rectal bleeding.
A
B
C
Supplementary Figure S1. Scoring of body weight change, stool consistency, and rectal bleeding. Colitis was induced in WT and VDUP1 KO mice by administering 2% DSS in the drinking water for 5 days followed by regular drinking water for an additional 4 days. Mice were evaluated daily for (A) body weight loss score, (B) stool consistency score, and (C)rectal bleeding score using indicated criteria (Supplementary Table S1) (n=9). § P <0.05, §§ P <0.01, for WT 2% DSS vs. VDUP1 KO 2% DSS, * P <0.05, ** P <0.01, *** P <0.001, **** P <0.0001 for control vs. 2% DSS.

## Slide 2
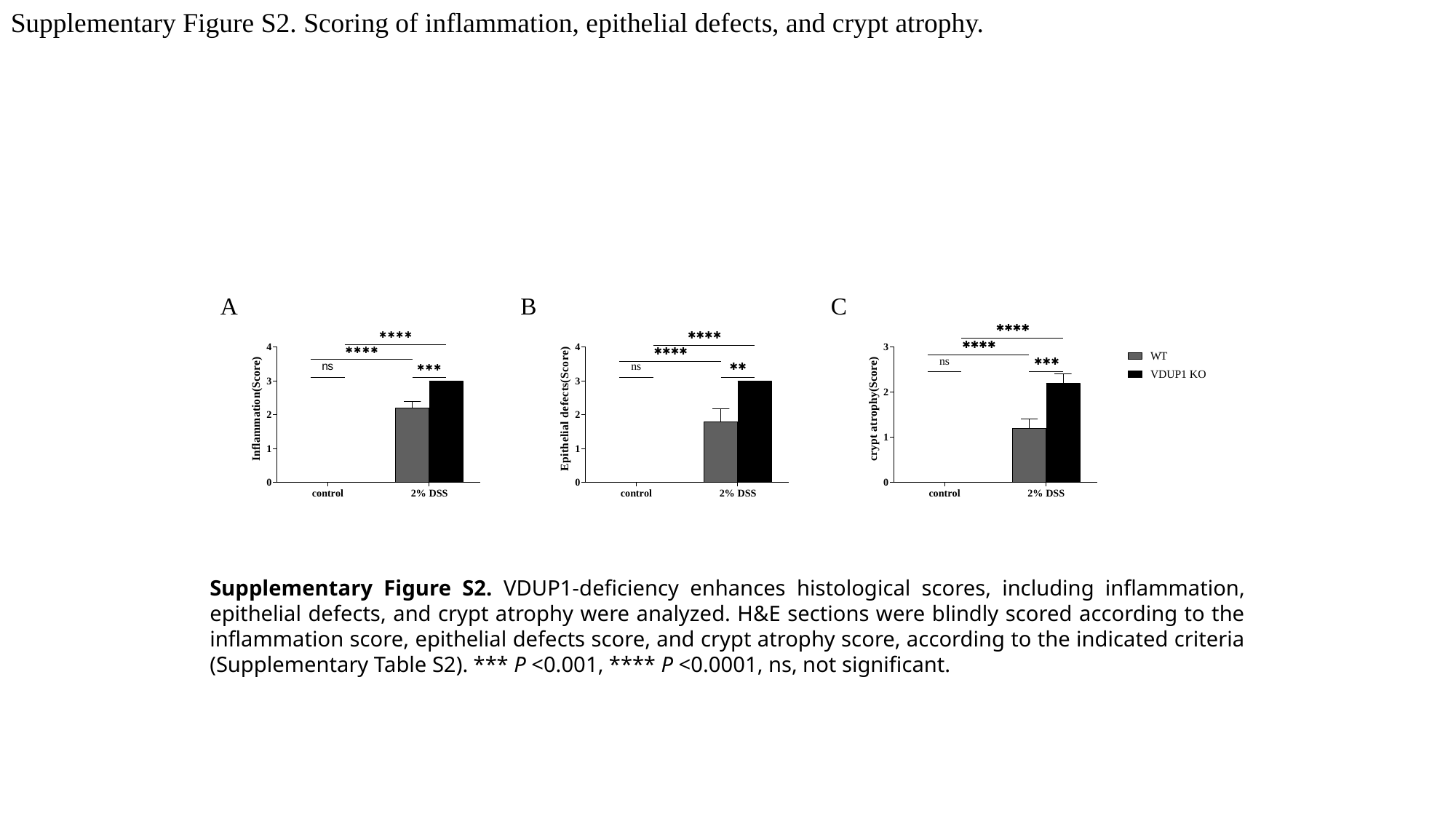

Supplementary Figure S2. Scoring of inflammation, epithelial defects, and crypt atrophy.
A
B
C
Supplementary Figure S2. VDUP1-deficiency enhances histological scores, including inflammation, epithelial defects, and crypt atrophy were analyzed. H&E sections were blindly scored according to the inflammation score, epithelial defects score, and crypt atrophy score, according to the indicated criteria (Supplementary Table S2). *** P <0.001, **** P <0.0001, ns, not significant.

## Slide 3
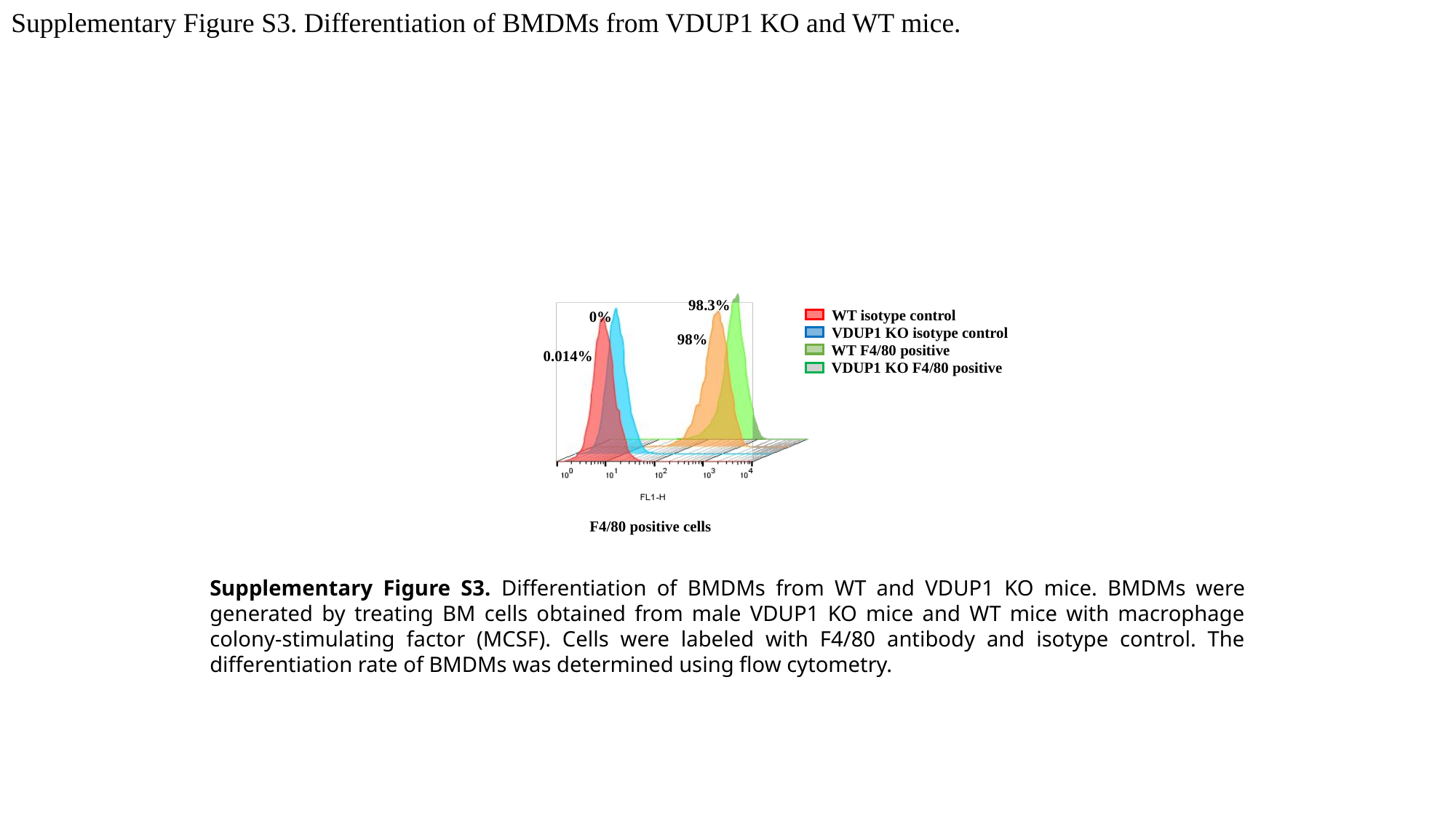

Supplementary Figure S3. Differentiation of BMDMs from VDUP1 KO and WT mice.
98.3%
WT isotype control
0%
VDUP1 KO isotype control
98%
WT F4/80 positive
0.014%
VDUP1 KO F4/80 positive
F4/80 positive cells
Supplementary Figure S3. Differentiation of BMDMs from WT and VDUP1 KO mice. BMDMs were generated by treating BM cells obtained from male VDUP1 KO mice and WT mice with macrophage colony-stimulating factor (MCSF). Cells were labeled with F4/80 antibody and isotype control. The differentiation rate of BMDMs was determined using flow cytometry.
