# Supplementary material for: VDUP1 Deficiency Promotes the Severity of DSS-Induced Colitis in Mice by Inducing Macrophage Infiltration
Source: Int J Mol Sci. 2023 Sep 1;24(17):13584. doi: 10.3390/ijms241713584 (PMC10487977; doi:10.3390/ijms241713584)
Supplement: Supplementary file 1 [file ijms-24-13584-s001.zip › Supplementary Tables_Final.pdf]

Supplementary Table S1. DAI scoring criteria.

Score	Body weight	Diarrhea Score	Rectal Bleeding
0	No weight loss	Normal pellets	Normal
1	Weight loss of 1-5%	Slightly loose feces	Slightly bloody
2	6-10%	Loose feces	Bloody
3	11-20%	watery diarrhea	growth bleeding
4	More than 20%		

Supplementary Table S2. Histological scoring criteria.

Score	Evaluation standard
	**Inflammation**
0	Normal
1	Small leukocyte aggregates in mucosa and/or submucosa
2	Coalescing mucosal and/or submucosal inflammation
3	Coalescing mucosal inflammation with prominent multifocal submucosal extension +/- follicle formation
4	Severe diffuse inflammation of mucosa, submucosa, & deeper layers
	**Epithelial defects**
0	None
1	Focally dilated glands and/or attenuated surface epithelium, decreased goblet cells
2	Focally extensive gland dilation and/or surface epithelial attenuation
3	Erosions (mucosal necrosis terminating above muscularis mucosae)
4	Ulceration (full-thickness mucosal necrosis extending into submucosa or deeper)
	**Crypt atrophy (in region most affected)**
0	None
1	<25%
2	~25—50%
3	~50—75%
4	>75%

Supplementary Table S3. Primer sequences for qRT-PCR.

Primer	Direction	Sequence (5′→3′)
β-actin	forword	TGGAATCCTGTGGCATCCATGAAAC
	reverse	TAAAACGCAGCTCAGTAACAGTCCG
IL-6	forword	AAAGAGTTGTGCAATGGCAATTC
	reverse	ATCTTTTACCTCTTGGTTGAAGATATGA
IL-1β	forword	TGTAATGAAAGACGGCACACC
	reverse	TCTTCTTTGGGTATTGCTTGG
TNF-α	forword	GCGGACTACTATGCTAAAGAGG
	reverse	GTAGAGTTCCACATGTTGCTCC
COX-2	forword	CGTGGTCACTTTACTACGAG
	reverse	AGGTACATAGTAGTCCTGAGC
VDUP1	forword	TGGCTCCAAGAAAGTCATCC
	reverse	TTGAGAGTCGTCCACATCGT
MUC2	forword	GCTGACGAGTGGTTGGTGAATG
	reverse	GATGAGGTGGCAGACAGGAGAC
F4/80	forword	CTTTGGCTATGGGCTTCCAGTC
	reverse	GCAAGGAGGACAGAGTTTATCGTG
CCL2	forword	AGGTCCCTGTCATGCTTCTG
	reverse	TCTGGACCCATTCCTTCTTG
CCL3	forword	CCTTGCTGTTCTTCTCTGTACCATG
	reverse	GCATTCAGTTCCAGGTCAGTGATG
KC	forword	TGCACCCAAACCGAAGTCAT
	reverse	TTGTCAGAAGCCAGCGTTCAC

Supplementary Table S4. Antibodies and serum used in this study.

Purchase from	Antibodies and Normal Serum	Host
	Immunohistochemistry	
MBL	VDUP1	Mouse
Abcam	F4/80	Rat
	Immunofluorescence	
Abcam	p-p65 (S536)	Rabbit
Vector Laboratories	Anti-Rabbit-IgG	Goat
